# Comparing the technical and individual possession statistics of academy players across different age groups in match-play

**DOI:** 10.1371/journal.pone.0316833

**Published:** 2025-01-13

**Authors:** Emily Escreet, Steve Barrett, John Toner, John Iga, Christopher Towlson

**Affiliations:** 1 Performance and Medicine Department, Norwich City, Norwich, United Kingdom; 2 School of Sport, Exercise and Rehabilitation Science, University of Hull, Hull, United Kingdom; 3 Playermaker, London, United Kingdom; Instituto Politecnico de Viana do Castelo, PORTUGAL

## Abstract

**Background:**

Youth soccer players in the UK transition into the professional game at 16 years of age. Understanding the differences between youth and professional standards can help coaches and clubs to support player development during this transition.

**Objectives:**

To (i) assess the differences in technical and possession statistics between different age groups (U16, U18, U23) and outfield positions (central defender [CD], wide defender [WD], central midfielder [CM], attacking midfielder [AM], wide midfielder [WM], striker [ST]), within an English academy soccer programme, during match-play.

**Methods:**

All matches were monitored using foot-mounted inertial measurements units (F-IMU; Playermaker^™^) to quantify the technical (touches and releases from the feet) and individual possession statistics (Time on the ball, Time on the ball per possession) from each match. Teams were instructed to play a 1-4-3-3 formation as part of their clubs playing philosophy, with positions defined per this formation. Data were analysed using a multi-variate ANOVA Two-tailed statistical significance was accepted as p ≤ 0.05 and measures of effect size were calculated using partial eta-squared (η^2^). Magnitude of the effect sizes were *small* (0.2<ES<0.6), *moderate* (0.6<ES<1.2), *large* (1.2<ES<2) and *very large* (≥2).

**Results:**

Trivial to small effect sizes were observed across age groups for both technical and possession-based statistics during match-play. Across all age groups and positions, CD`s had the highest number of technical actions (touches and releases), whereas ST`s & AM`s, had the highest amount of time in possession of the ball. Further, positional analysis showed ST`s and AM`s had moderate to large decreases in time on the ball per possession at the U23’s age group in comparison to the U16 and U18’s, with no technical differences observed within the same positional analysis.

**Conclusion:**

Attacking players (AM & ST) are required to move the ball quicker as they progress from U16 to U23’s within the current English football academy. Further exploration is required to assess if these changes are context specific given requirements of those age groups.

## Introduction

Youth development programmes are in place to support a clear pathway for talented athletes/ players to perform at the highest level [1]. Within soccer, the transition from being a part-time youth soccer player to a full-time, (available for senior professional selection) is desirable for soccer players and coaches to succeed and subsequently satisfy financial fair play regulations (UEFA). In an attempt to satisfy such regulation, many European professional soccer leagues have introduced long-term athletic and talent development frameworks, with the objective to enhance the number of ‘home-grown’ soccer players being available. In England, the Elite Player Performance Plan (EPPP) was introduced by the Premier League/ English FA to support and standardise operations across clubs to ensure youth development pathways provided the “best” environment for players to succeed in their objectives for becoming a professional [2, 3]. As part of this, clubs are required to support and monitor players with insights into their development including (but not limited to), physical and technical performances of training and matches [1]. These insights allow clubs to create technical performance benchmarks across age groups in order to identify talented players within their club [4].

Time-motion analysis (such as running speeds and total distance covered) and notational analysis (representing technical outputs e.g. passes, shots, tackles etc.) player data are utilised to help inform selection processes by coaches and practitioners [5]. While a plethora of data exists reporting time-motion analysis metrics derived from global positioning systems (GPS) [6, 7], technical data is in general reported only within senior matches [8, 9], due to the manual coding required to obtain such metrics [10], within youth soccer. This process requires a significant amount of human resource and can result in significant measurement error owing the inherent subjective nature of observing and then interpreting performance against pre-determined operational definitions [11]. The minimal research that has been undertaken within an academy setting has utilised semi-automatic tracking systems [12], however, the financial investment required hinders the repeatability and transferability to other academy cohorts [1]. A potential cost effective and objective solution to monitoring technical actions within match play is through the use of wearable microtechnology [10].

Both training and match-play insights are valuable to key stakeholders [13], to understand how players are developing, given that youth players performance can vary due to multiple factors including, stage of development [14, 15], psychological stresses (i.e. social challenges, school exams;), transition between part-time to full-time player [3, 16], increased external pressures [17]. Moreover, preparing players for the next stage in their development is a pertinent area of consideration for practitioners [18]. Players are required to meet the match demands of the next level in order to be successful [19]. However, achieving this requires a clear understanding of the technical skills needed at each level, particularly across various age groups and playing positions. Given the lack of empirical evidence, further research is essential within academy match play. Quantifying technical actions within match play would provide a broader understanding of not only individual development but also the difference in positional demands [20]. At a senior level it has been reported that central attacking midfielders attain the highest amount of ball possessions (57 ± 8) and touches per possession (2.24 ± 0.45) with central defenders having the least (41 ± 10 and 1.74 ± 0.39) [21]. However, due to the study being conducted over the course of the 2006–2007 season, it could be suggested that the findings aren’t applicable to modern day football. Previous research has shown that the frequency of ball touches and passes increased by 10.5% and 29.9% respectively, over the course of seven seasons [22], highlighting the need for current research.

The application of foot-mounted inertial measurement units (F-IMUs) has enabled the objective collection of technical data across a range of competitive environments, providing new insights into player performance [5, 10, 13]. With academy soccer coaches emphasising the importance of technical metrics, such as possession and ball handling statistics, to help identify and develop talented soccer players [4]. However, despite the growing availability of physical performance data [23, 24], there remains a critical need for comprehensive technical data to inform training and development strategies across different age groups and playing positions [25]. Therefore, the objective of this study was to compare the technical and possession statistics from match play between age groups and positions within a professional English soccer club.

## Methods

### Participants

A total of fifty-two academy football players at U16 (15.2 ± 0.6 years, height 179.4 ± 6.4 cm, weight 69.2 ± 11.4 kg), U18 (17.1 ± 0.5 years, height 181.7 ± 8.3 cm, weight 78.1 ± 8.5 kg) and U23 (18.9 ± 0.7 years, height 183.1 ± 6.8 cm, weight 74.7 ± 5.7 kg), from a category 1 football academy [5, 26], voluntarily enrolled in this study. Players were recruited between October 1^st^ 2021 to May 7^th^ 2022. Informed consent was gained from the players and the parents if required. The inclusion criteria were regular participation in the academy football programme. The study attainted ethical approval (FHS368), with data collected as part of the players normal match day monitoring.

### Experimental design

Technical actions were quantified in the U16, U18 and U23 age group across the 2021–22 football season. All matches (U23 = 28, U18 = 34, U16 = 18) were played as part of the Premier League fixtures. Pre-Season games were excluded from the study. The U16 and U18 age groups played with 11 players and utilised the “rolling substitute”, with the U23 age groups being limited to 3 substitutions per game. A total of 554 positional observations comprised of goalkeepers (U23 = 14, U18 = 20, U16 = 4), full backs (U23 = 96, U18 = 42, U16 = 15), centre backs (U23 = 52, U18 = 53, U16 = 20), centre midfield (6) (U23 = 17, U18 = 20, U16 = 7), centre midfield (8) (U23 = 34, U18 = 20, U16 = 13), attacking midfield (10) (U23 = 23, U18 = 30, U16 = 5) and strikers (U23 = 46, U18 = 39, U16 = 21) were included. Playing positions were determined by the lead coach of each age group with a playing philosophy of 4-3-2-1 remaining constant throughout.

Players were required to have played the full duration of the match with U23 and U18 squads match duration being 90 minutes and U16 squad 80 minutes. A total of 80 matches were included in the data set for analysis which included 554 player observations. Observations that contained missing data due to technological error were removed from the analysis. Pre-Season games were excluded from the study.

### Inertial measurement units

Locomotor activities, technical actions and individual possession statistics were quantified using commercially available F-IMUs (PlayerMaker^™^, Tel Aviv, Israel). Previous research has demonstrated validity and reliability of the units [10, 13, 23]. Each F-IMU incorporated two components from the MPU-9150 multi-chip motion tracking module (InvenSense, California, USA), being a 16 g triaxial accelerometer and a 2000°•sec^-1^ triaxial gyroscope. Housed in manufacturer-supplied tightly fitting silicone straps, each player was equipped with two IMUs (one for each foot), which were located at the lateral malleoli over the player’s boots. To diminish issues related to inter-unit reliability, players used the same F-IMUs throughout the data collection period [27, 28]. All devices were activated via a Bluetooth connection to an iPad (Apple Inc, California) prior to each match. Data were uploaded to the manufacturer’s cloud-based software (v.3.22.0.02) post-match by the club practitioners. The start and end of each match was identified and tagged prior to data being exported from the manufacturer’s cloud-based software into Microsoft Excel 2020.

### Technical characteristics

Technical actions were defined as the number of releases and the number of touches [10], that a player had within a match situation. ouches refer to any instance where the foot makes contact with the ball, while releases refer to any instance where the foot strikes the ball, including actions such as passes, crosses, shots, and clearances [29]. High speed ball releases (>15m/s) [13] where also monitored.

### Individual possession statistics

Within technical actions, an individual player possession was defined as, the time between a player receiving a ball and releasing the ball [30]. This definition was used to quantify the total time a player spent on the ball within a game and their average time on the ball per an individual possession. Possessions where further split into one-touch (OT), short-possession (<1.5s; SP) and long-possessions (>1.5s; LP). While the number of touches, releases and high-speed releases have been shown to be valid and reliable [10, 13], the time between a touch and a release have not yet been defined within the current literature. As such these reports are preliminary with recommendations made to assess the validity and reliability of these metrics against a criterion measure. However, the authors where unable to find an objective criterion measure for time on the ball.

### Statistical analysis

Prior to the analysis, Q-Q plots and stem and leaf charts were monitored to check for normal distribution. Assumptions of normality were further assessed by plotting boxplots of the residuals and a scatterplot of the predicted values. A linear mixed model was then used to assess the differences of the technical (Touches, Releases, High-Speed Releases) and Possession statistics (Total time on the ball, Time on the ball per possession) between each age group. General linear models were able to account for the different number of samples between age groups. Post-hoc pairwise comparisons, with Sidak adjusted *p* values, were conducted in the event of a statistically significant F-ratio. Analyses were completed using IBM SPSS Statistics for windows software (release 20; SPSS Inc., Chicago, IL, USA). Two-tailed statistical significance was accepted as p ≤ 0.05 and measures of effect size were calculated using partial eta-squared (η^2^). Magnitude of the effect sizes were *small* (0.2<ES<0.6), *moderate* (0.6<ES<1.2), *large* (1.2<ES<2) and *very large* (≥2).

## Results

### Age group

No significant differences were found between age groups for each of the technical variables (Table 1).

**Table 1:** Estimated marginal mean values representative of the average time on ball and the relative frequency of ball touches and releases per minute of match duration, performed by professional soccer players in a match.

Variable	U23			U18			U16		
	EM Mean	SE	95% CI	EM Mean	SE	95% CI	EM Mean	SE	95% CI
**Avg Time on Ball (sec)**	1.133	0.028	1.078–1.188	1.197	0.026	1.147–1.247	1.136	0.049	1.039–1.233
	U18^T^, U16^T^			U23^T^, U16^T^			U23^T^, U18^T^		
**Releases (ƒ.min-1)**	0.441	0.011	0.419–0.462	0.419	0.010	0.399–0.439	0.422	0.019	0.384–0.460
	U18^T^, U16^S^			U23^T^, U16^T^			U23^S^, U18^T^		
**Ball Touches (ƒ**.**min-1)**	1.034	0.026	0.983–1.085	1.024	0.024	0.977–1.071	0.988	0.045	0.899–1.077
	U18^T^, U16^T^			U23^T^, U16^T^			U23^T^, U18^T^		

**N.B.** EM = estimated marginal. SE = standard error. CI = confidence intervals. Statistically significant differences (p ≤ 0.005) are depicted in bold: Observed magnitude of effects are denoted as: [^T^] = trivial; [^S^] = small; [^M^] = moderate; [^L^] = large; and [^V^] = very large.

### Playing position

STs and AM(10)s (1.66 ± 0.04 and 1.40 ± 0.07, 95% CI; 1.58–1.73 and 1.27–1.53, ES;1.28 to 2.03 [Large to Very Large] and 0.58 to 1.28 [Small to Large], P ≤ 0.019 and P ≤ 0.019) had significantly greater average ball time compared to all other positions, with STs having the longest average time. GKs (0.77 ± 0.08, 95% CI; 0.62 to 0.92, ES; 0.58 to 2.03, P ≤ 0.001) had significantly lower average time on ball compared with CD, CM(8), AM(10) and ST, however, showed no clear difference between WD and CM(6). ST and AM(10) had significantly fewer releases/min in comparison to other positions, with CD having significantly more. Goalkeepers had significantly fewer touches/min compared to CD, WD, CM(6) and CM(8), and there was no clear difference compared to AM(10) and ST (Table 2).

**Table 2:** Estimated marginal mean values representative of the inter-positional differences in average time on ball and the relative frequency of ball touches and releases per minute of match duration, performed by professional soccer players in a match.

Variable	GK			CB			FB			CM (6)			CM (8)			AM			ST		
	EM Mean	SE	95% CI	EM Mean	SE	95% CI	EM Mean	SE	95% CI	EM Mean	SE	95% CI	EM Mean	SE	95% CI	EM Mean	SE	95% CI	EM Mean	SE	95% CI
**Avg Time on Ball (sec)**	0.771	0.077	0.621–0.921	1.101	0.037	1.028–1.174	1.036	0.043	0.952–1.120	0.998	0.063	0.875–1.122	1.129	0.046	1.039–1.218	1.398	0.066	1.268–1.529	1.655	0.039	1.579–1.731
	**CB**^**M**^, FB^S^, CM (6)^S^, **CM (8)**^**M**^, **AM**^**L**^, **ST**^**VL**^			**GK**^**M**^, FB^T^, CM (6)^S^, CM (8)^T^, **AM**^**M**^, **ST**^**L**^			GK^S^, CB^T^, CM (6)^T^, CM (8)^S^, **AM**^**M**^, **ST**^**L**^			GK^S^, CB^S^, FB^T^, CM (8)^S^, **AM**^**M**^, **ST**^**L**^			**GK**^**M**^, CB^T^, FB^S^, CM (6)^S^, **AM**^**S**^, **ST**^**L**^			**GK**^**L**^, **CB**^**M**^, **FB**^**M**^, **CM (6)**^**M**^, **CM (8)**^**S**^, **ST**^**S**^			**GK**^**VL**^, **CB**^**L**^, **FB**^**L**^, **CM (6)**^**L**^, **CM (8)**^**L**^, **AM**^**S**^		
**Releases (ƒ.min-1)**	0.483	0.030	0.424–0.543	0.634	0.015	0.605–0.663	0.451	0.017	0.417–0.484	0.502	0.025	0.453–0.551	0.402	0.018	0.367–0.438	0.301	0.029	0.245–0.358	0.228	0.015	0.198–0.258
	**CB**^**M**^, FB^T^, CM (6)^T^, CM (8)^S^, **AM**^**M**^, **ST**^**L**^			**GK**^**M**^, **FB**^**M**^, **CM (6)**^**M**^, **CM (8)**^**L**^, **AM**^**L**^, **ST**^**VL**^			GK^T^, **CB**^**M**^, CM (6)^S^, CM (8)^S^, **AM**^**M**^, **ST**^**L**^			GK^T^, **CB**^**M**^, FB^S^, **CM (8)**^**S**^, **AM**^**M**^, **ST**^**L**^			GK^S^, **CB**^**L**^, FB^S^, **CM (6)**^**S**^, **AM**^**M**^, **ST**^**M**^			**GK**^**M**^, **CB**^**L**^, **FB**^**M**^, **CM (6)**^**M**^, **CM (8)**^**M**^, ST^S^			**GK**^**L**^, **CB**^**VL**^, **FB**^**L**^, **CM (6)**^**L**^, **CM (8)**^**M**^, AM^S^		
**Ball Touches (ƒ**.**min-1)**	0.788	0.071	0.649–0.927	1.285	0.035	1.217–1.353	1.030	0.040	0.953–1.108	1.172	0.058	1.057–1.286	1.046	0.042	0.963–1.129	0.965	0.067	0.833–1.096	0.847	0.036	0.776–0.917
	**CB**^**L**^, **FB**^**S**^, **CM (6)**^**M**^, **CM (8)**^**M**^, AM^S^, ST^T^			**GK**^**L**^, **FB**^**M**^, CM (6)^S^, **CM (8)**^**M**^, **AM**^**M**^, **ST**^**M**^			GK^S^, **CB**^**M**^, CM (6)^S^, CM (8)^T^, AM^S^, **ST**^**S**^			**GK**^**M**^, CB^S^, FB^S^, CM (8)^S^, AM^S^, **ST**^**M**^			**GK**^**M**^, **CB**^**M**^, FB^T^, CM (6)^S^, AM^S^, **ST**^**S**^			GK^S^, **CB**^**M**^, FB^S^, CM (6)^S^, CM (8)^S^, ST^S^			GK^T^, CB^M^, FB^S^, CM (6)^M^, CM (8)^S^, AM^S^		

**N.B.** EM = estimated marginal. SE = standard error. CI = confidence intervals. Statistically significant differences (p ≤ 0.005) are depicted in bold: **GK** = Goalkeepers; **CB** = Centre Backs; **FB** = Full Backs; **CM (6)** = Centre Midfield (6); **CM (8)** = Centre Midfield (8); **AM** = Attacking Midfield; **ST** = Striker. Observed magnitude of effects are denoted as: [^T^] = trivial; [^S^] = small; [^M^] = moderate; [^L^] = large; and [^V^] = very large.

### Position between age groups

**Fig 1:**
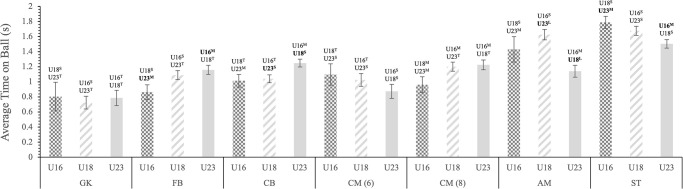
Estimated marginal mean (± SE) age group differences in the average time on ball performed by professional soccer players on a match day for each position. Each bar represents an age group. Statistically significant differences (p ≤ 0.005) are displayed above SE bars and are depicted in bold. Observed magnitude of effects are denoted as: [^T^] = trivial; [^S^] = small; [^M^] = moderate; [^L^] = large; and [^V^] = very large.

**Fig 2:**
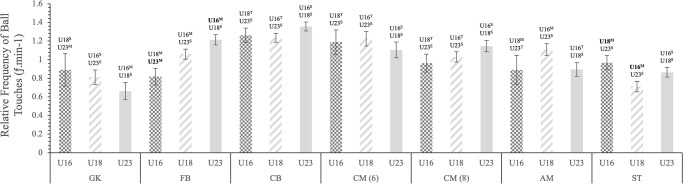
Estimated marginal mean (± SE) age group differences in the relative frequency of ball touches per minute, performed by professional soccer players on a match day for each position. Each bar represents an age group. Statistically significant differences (p ≤ 0.005) are displayed above SE bars and are depicted in bold. Observed magnitude of effects are denoted as: [^T^] = trivial; [^S^] = small; [^M^] = moderate; [^L^] = large; and [^V^] = very large.

**Fig 3:**
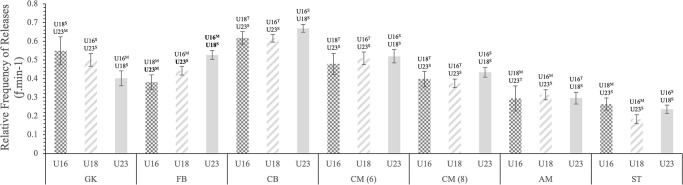
Estimated marginal mean (± SE) age group differences in the relative frequency of releases per minute, performed by professional soccer players on a match day for each position. Each bar represents an age group. Statistically significant differences (p ≤ 0.005) are displayed above SE bars and are depicted in bold. Observed magnitude of effects are denoted as: [^T^] = trivial; [^S^] = small; [^M^] = moderate; [^L^] = large; and [^V^] = very large.

There was no significant difference in average ball time between age groups for GKs, CM(6) and CM(8), however, a significant difference was found between the U16s and U23s in both ST and WD, and between U18s and U23s in both AM(10) and CD (Fig 1). This trend continued for GKs, CM(6) and CM(8) with the addition of CD and AM(10), showing no significant difference between the age groups in releases/min. Although significant differences were found between the U16 and U23 WDs and U16 and U18 STs (Fig 2). No significant differences were found in touches/min between age groups for all positions expect WD, in which U16 had less touches/min compared to U18 and U23 (Fig 3).

## Discussion

The purpose of this study was to compare the difference between age groups and positions across technical and possession-based statistics in a professional soccer academy, using foot-mounted F-IMU’s. The main results were three-fold, i) that trivial to small differences were observed across all technical and possession based statistics between U16, U18 and U23 match-play; ii) CD`s had the highest number of technical actions (touches and releases) during match-play; while ST`s and AM`s had the most number of possessions; iii) ST`s and AM`s players spent less time on the ball on average per possession at the U23’s age group in comparison to the U18 and U16’s.

Youth development programmes within professional soccer clubs, aim to produce players capable of playing in their first team [3, 31]. As players progress through an academy, key performance indicators will adjust to represent the demands of match-play for that age group, with transitioning into first team football the main aim [3, 31]. The current study explored the progression of technical and possession-based statistics using foot-mounted IMU’s to monitor academy match-play, showing trivial to small effects across age groups on average. Soccer clubs, in order to support players progression to the first team, have started to build philosophies and playing identities, which are filtered throughout the club by coaching staff [17]. Within this exploratory analysis of a single club, technical and possession-based statistics were similar, which maybe reflective of a clear playing style and requirements of the age groups to play the clubs philosophy. As the current study examined an individual club, further research is required to understand if these trends are transferable across academies of different levels and in different countries. Further differences may exist when players are required to play out on loan at lower-level professional or semi-professional team, where playing/ training philosophy of the club could be different to that of the parent club [32]. Other considerations within the current study are that 2 of the age groups (U23 & U18) were full-time programmes, and 1 a part-time school boy age group (U16). With findings similar during match-play across age groups (Table 1), differences maybe more evident within a training environment as opposed to in match-play. With coaching programmes designed to optimally prepare players for the requirements of match-play, further research should examine the differences within training and match technical and possession-based statistics.

Technical and possession-based statistics have been well documented at senior team level using semi-automated tracking systems or manual coding processes [20]. Within youth level programmes, less information is available due to the manual coding processes required and human resources of clubs to automate some of these insights [9, 10]. This study, to the authors knowledge, was the first to observe positional differences across male academy level age groups using F-IMU`s in match-play. A novel finding from the study, identified that both ST`s and AM`s have the greatest number of possessions, but spend less time on the ball when they are in possession at the U23 age group in comparison to the other age groups (on average moving the ball quicker, when they receive the ball). To break oppositions defences down, attacking players may be required to move the ball quicker [33]. While style of play may be influenced at first team level by a number of contextual factors such as opposition style of play, match location or league positioning [33], this information appears unavailable at academy level, and may have further implications for the findings of these results. For example, it has been identified that when certain teams played a certain style of play, an opponent would adopt another style of play to challenge that team [33]. With player development a key focus of academy football [15], this may not be relevant for the current population, with further research required to gain a greater understanding of how contextual factors influence academy teams playing style.

Professional level soccer becomes increasingly reliant on a positive result for players and staff to be successful within their roles, whereas at academy level football, the aim is to develop players for the first team [2, 3, 17]. Further exploration is warranted to better understand what the objectives are for each player, in each position, at each age group, to better understand if the differences in time spent on the ball per possession because of this. Greater tactical awareness/understanding of the game has been evidenced in senior players in comparison to younger players [1]. While beyond the scope of the current study, further examination of the coaching process and the multi-disciplinary teams’ objectives for a given age group, should be considered within technical and possession-based statistics in youth soccer.

### Practical implications

The results of this study allow coaches to have a tailored strategy toward player development. Training sessions could be designed with technical demands which are dependent on a player’s specific position. This is also applicable at specific age groups with average time on ball decreasing as the player’s go through the development pathway. The finding that technical and possession based statistics are similar across age groups might reflect a consistent playing philosophy, and ensuring coaching staff across all age groups adhere to this philosophy can facilitate a smoother transition for players as they progress through the academy. The implementation of foot-mounted IMUs across age groups has been shown to provide consistent and objective data for monitoring player performance. In this context it can be a cost effective and time efficient alternative over existing technologies.

### Limitations

The study was conducted within a single football club, which means that the findings may not be applicable to other clubs with different playing styles, training philosophies or competition levels. There is also a lack of contextual factors such as the quality of the opposition, match location, or specific game situations which might influence player performance and therefore technical and possession based actions. Further research is needed across a wide range of clubs whilst also accounting for contextual factors.

## Conclusions

This investigation has provided a novel understanding of the difference in technical match play demands at an age group level and also between positions. The findings suggest that was players progress through the academy, the technical and possession based demands of match- play remain relatively consistent, reflecting a clear and consist playing philosophy within the club. The positional differences highlight the unique demands placed on players in different roles, providing valuable insights for tailored coaching strategies. Although the study does have its limitations it still provides an understanding of technical and possession-based performance in youth soccer and underscores the importance of consistent playing philosophies and tailored coaching strategies. Future research should explore these trends across multiple clubs and contexts, consider longitudinal approaches, and integrate a broader range of performance indicators, including psychological and contextual factors, to provide a more comprehensive understanding of player development in soccer academies.
